# Immediate-Type PPI Hypersensitivity as a Severe Adverse Drug Reaction: Diagnostic Challenges, Cross-Reactivity, and Real-Life Outcomes

**DOI:** 10.3390/jcm15051808

**Published:** 2026-02-27

**Authors:** Ragıp Fatih Kural, Ceyda Tunakan Dalgıç, Yusuf Özeke, Kasım Okan, Salih Afşin, Reyhan Gümüşburun, Kutay Kırdök, Züleyha Galata, Meryem İrem Toksoy Şentürk, Ümitcan Ateş, Eda Aslan, Emine Nihal Mete Gökmen, Aytül Zerrin Sin

**Affiliations:** 1 Division of Immunology and Allergy, Department of Internal Medicine, Faculty of Medicine, Ege University, 35100 İzmir, Türkiye; ragip.fatih.kural@ege.edu.tr (R.F.K.); yusuf.ozeke@ege.edu.tr (Y.Ö.); kasimokan55@gmail.com (K.O.); reyhangumusburun@gmail.com (R.G.); kirdokkutay@gmail.com (K.K.); zuleyhagalata61@gmail.com (Z.G.); irem.toksoy@yahoo.com (M.İ.T.Ş.); umitcanates@gmail.com (Ü.A.); edaarslan_91@hotmail.com (E.A.); enihalmete@yahoo.com.tr (E.N.M.G.); aytulsin@yahoo.com (A.Z.S.); 2 Department of Internal Medicine, Faculty of Medicine, Ege University, 35100 İzmir, Türkiye; salih.afsin@ege.edu.tr

**Keywords:** adverse drug reaction, cross-reactivity, drug hypersensitivity, drug provocation test, drug safety, pharmacovigilance, proton pump inhibitors, real-world evidence

## Abstract

**Background/Objectives**: Immediate-type hypersensitivity reactions (HSRs) to proton pump inhibitors (PPIs) represent a clinically relevant adverse drug reaction and pose diagnostic and management challenges, including variable cross-reactivity (CR). Drug provocation testing (DPT) is the diagnostic gold standard, but real-life use after testing remains uncertain. This study aimed to describe clinical features, diagnostic outcomes, CR profiles, and real-life PPI use in patients with immediate-type PPI hypersensitivity. **Methods**: Single-center ambispective study of 40 patients evaluated for immediate-type PPI HSRs (2014–2023). Index reactions, culprit PPI, and skin test/DPT results were recorded retrospectively. Patients tolerating an alternative PPI on DPT were followed prospectively; real-life tolerance and use were assessed by structured telephone interview. **Results**: Among 40 patients (87.5% female; mean age 48 years), lansoprazole was the most frequent culprit (50%); anaphylaxis occurred in 65%. Skin tests were positive in 25% (*n* = 9), and a shorter interval from reaction to testing was associated with positivity (*p* = 0.025). CR was detected in 25% (*n* = 10), most often between lansoprazole and pantoprazole. All 29 patients undergoing DPT tolerated at least one alternative PPI. In real-life follow-up (*n* = 27), 11 (40.7%) restarted PPIs without recurrence; 1 (3.7%) developed a mild cutaneous reaction despite negative testing. Fifteen (55.6%) did not restart therapy; 4 (14.8%) cited drug-related anxiety or physician-advised avoidance. **Conclusions**: In immediate-type PPI hypersensitivity, skin test sensitivity appears time-dependent, supporting early evaluation. DPT identifies safe alternatives, yet behavioral and clinician-related barriers limit real-life implementation; addressing these barriers is essential to optimize PPI allergy management.

## 1. Introduction

Proton pump inhibitors (PPIs) are widely prescribed worldwide because of their high efficacy in the management of acid-related gastrointestinal disorders. These medications are generally considered safe, with adverse reactions reported in only 1–3% of users. However, given their extensive worldwide use, even rare adverse drug reactions (ADRs) may affect a substantial number of patients [1]. Despite their favorable safety profile, hypersensitivity reactions (HSRs) to PPIs are increasingly recognized and represent a clinically relevant ADR due to their potential severity, diagnostic complexity, and implications for subsequent prescribing decisions.

PPI-induced HSRs are generally classified into two main groups: immediate-type reactions, typically driven by Immunoglobulin E (IgE)-mediated mechanisms, and delayed-type reactions, which are T-cell-mediated [2]. Immediate-type reactions range in severity from cutaneous manifestations, such as urticaria and angioedema, to life-threatening anaphylaxis involving the respiratory and cardiovascular systems [3,4,5].

The PPI class consists of seven different agents: omeprazole, pantoprazole, esomeprazole, lansoprazole, rabeprazole, dexlansoprazole, and tenatoprazole. PPIs share a common chemical structure composed of benzimidazole and pyridine rings. Generally, omeprazole, esomeprazole, and pantoprazole differ by substitutions on the benzimidazole ring, whereas lansoprazole, rabeprazole, and dexlansoprazole differ by modifications on the pyridine ring [6,7]. These structural characteristics not only dictate pharmacological properties but also underlie the cross-reactivity (CR) patterns observed in immediate-type HSRs [8].

Diagnostic algorithms recommended by the European Academy of Allergy and Clinical Immunology (EAACI) advocate the systematic use of skin prick tests (SPT), intradermal tests (IDT), and drug provocation tests (DPT) for the evaluation of PPI hypersensitivity. Skin tests (ST) demonstrate high specificity but limited sensitivity. Furthermore, a prolonged interval between the reaction and testing may further reduce diagnostic sensitivity. In addition, the applicability of these algorithms is markedly limited because IDT cannot be performed with oral formulations and intravenous preparations are not available for certain PPIs. For these reasons, DPT is considered the gold standard for confirming the diagnosis in patients with persistent clinical suspicion, even when skin tests are negative. Additionally, DPT allows for the assessment of CR among PPIs and the identification of safe alternative agents, thereby supporting risk mitigation and continuity of therapy [2].

Since studies on PPI hypersensitivity are predominantly based on limited patient series, the current literature is constrained not only by a scarcity of epidemiological data but also by fundamental diagnostic and pathophysiological uncertainties. Major knowledge gaps include the undefined relationship between chemical structures and CR patterns, as well as the lack of identified antigenic determinants. Furthermore, reliable in vitro tests, such as specific IgE or validated basophil activation tests, remain unavailable for confirming the diagnosis without the risks of DPT. However, beyond these diagnostic limitations, one of the most critical practical gaps is the uncertain ability of diagnostic tests to predict real-life tolerance. From a clinical pharmacology perspective, the real-world translation of diagnostic test results into routine prescribing practice remains unclear. In other words, whether a PPI identified as ‘safe’ under controlled conditions maintains the same safety and is actually reintroduced in real-life use represents the central clinical question of this study.

To address this gap, we designed the present study based on a patient cohort evaluated at our center. Our primary objective was to describe the clinical characteristics of immediate-type PPI HSRs and the distribution of culprit agents. Secondarily, we assessed diagnostic test results and CR profiles. Finally, we aimed to determine the practical predictive value of these tests by analyzing the real-life safety and use of PPIs identified as ‘safe’ during the diagnostic work-up.

## 2. Materials and Methods

### 2.1. Study Design and Participants

This single-center ambispective (retrospective–prospective) study was conducted at the Division of Immunology and Allergy, Department of Internal Medicine, Ege University Faculty of Medicine.

In the retrospective phase, we analyzed data from 40 patients who presented with a history of immediate-type HSRs to PPIs between 2014 and 2023 and completed the drug allergy work-up. The inclusion criterion was a history of suspected immediate-type adverse drug reactions consistent with HSRs following PPI administration. Patients with incomplete medical records, an unclear temporal relationship between drug administration and reaction onset, or a history of delayed-type reactions were excluded.

The study was approved by the Medical Research Ethics Committee of Ege University Faculty of Medicine (Decision No: 25-2T/2; 6 February 2025). Verbal informed consent was obtained from all participants for the structured telephone interviews conducted as part of real-life data collection. All procedures were conducted in accordance with the Declaration of Helsinki and relevant institutional guidelines.

### 2.2. Clinical Data and Index Reaction Characteristics

Using a standardized form, we recorded demographic details including age, sex, history of atopy, and comorbidities, as well as the culprit PPI and the specific symptoms and systems involved in the HSRs. Reaction severity was classified according to the Brown Anaphylaxis Grading System: Grade 1 (limited to skin and subcutaneous tissues), Grade 2 (features suggesting respiratory, cardiovascular, or gastrointestinal involvement), and Grade 3 (hypoxia, hypotension, or neurological compromise) [9]. The time to onset of symptoms following PPI administration and the interval between the index reaction and diagnostic testing were also analyzed to assess factors influencing diagnostic yield.

### 2.3. Drug Allergy Work-Up

All diagnostic procedures were performed by an experienced allergist using concentrations established as non-irritant in the literature [4,5]. Emergency resuscitation equipment was readily available throughout the procedures to ensure patient safety during ADR evaluation.

#### 2.3.1. Skin Tests

SPTs were performed using commercial oral preparations containing omeprazole (20 mg), lansoprazole (30 mg), pantoprazole (40 mg), rabeprazole (20 mg), and esomeprazole (20 mg). Tablets or capsules were crushed using a mortar and dissolved in 1 mL of 0.9% sterile saline. This procedure yielded final concentrations (20 mg/mL for omeprazole and esomeprazole, 30 mg/mL for lansoprazole, 40 mg/mL for pantoprazole, and 20 mg/mL for rabeprazole) that remained well within the maximum non-irritant SPT limits (e.g., 40 mg/mL for omeprazole and esomeprazole) recommended by the EAACI position paper [2]. To ensure stability and prevent degradation due to potential pH instability, these solutions were prepared immediately prior to skin testing. The suspensions were manually homogenized until a visually uniform solution was obtained and were used immediately after preparation to minimize variability in drug concentration and potential degradation. No stabilizing or buffering agents were added.

Additionally, injectable forms of omeprazole (4 mg/mL), pantoprazole (4 mg/mL), and esomeprazole (8 mg/mL) were used at 1:10 and 1:1 dilutions for SPT when available.

The tests were applied to the volar surface of the forearm with positive (histamine) and negative (saline) controls and evaluated after 20 min. A wheal diameter of ≥3 mm compared to the negative control was considered positive.

To ensure procedural standardization and to avoid potential irritant responses associated with oral preparations, IDT was performed exclusively with injectable formulations. IDTs were performed by intradermally injecting 0.03 mL of test solution using injectable forms of omeprazole, pantoprazole, and esomeprazole at dilutions of 1:1000, 1:100, and 1:10. Stepwise dilutions were applied sequentially, starting from the lowest concentration, and testing was discontinued if a positive response occurred at any stage. After 15 min, an increase in wheal diameter of ≥3 mm compared to the initial wheal, accompanied by erythema, was considered positive.

#### 2.3.2. Drug Provocation Tests

Single-blind, placebo-controlled DPTs were performed in patients with negative skin test results to confirm the diagnosis or to identify safe alternative PPIs for future use.

Testing began with administration of a placebo capsule containing starch, followed by stepwise incremental doses of the active drug until the full therapeutic dose was achieved or objective clinical findings occurred. Dose escalation was performed at 30 min intervals.

Doses were titrated in steps of 5–10–20 mg for omeprazole, pantoprazole, rabeprazole, and esomeprazole, and in steps of 7.5–15–30 mg for lansoprazole. The test was considered positive if objective signs resembling the index reaction (e.g., urticaria, angioedema, respiratory symptoms) developed. Subjective symptoms without accompanying objective clinical signs were not considered sufficient for test positivity.

Patients were observed for at least three hours after the last dose to capture immediate ADRs. This extended observation period was applied due to the delayed absorption characteristics of slow-release PPI formulations.

### 2.4. Definition of Cross-Reactivity Groups

Upon completion of the drug allergy work-up, potential cross-reactivity (CR) among PPIs was assessed, taking into account cases confirmed by skin testing or DPT, as well as those based solely on clinical history. Test-confirmed CR was defined as positive results in skin tests (SPT and/or IDT) or DPTs to more than one PPI. Clinical CR was defined as recurrent immediate HSRs to two or more PPIs in patients for whom cross-sensitivity could not be objectively confirmed by skin tests or DPTs.

### 2.5. Prospective Follow-Up and Assessment of Real-Life Tolerance

In the prospective phase, patients for whom a safe alternative PPI was identified were followed up via structured telephone interviews. Patients were asked about their real-life use of the drug, the occurrence of any reactions, and the reasons for non-use despite negative diagnostic testing. The number of patients who could not be contacted and the reasons were recorded. In cases where a reaction occurred, the timing (after the first or repeated doses), type, and severity were also noted.

### 2.6. Statistical Analysis

Statistical analyses were performed using IBM SPSS Statistics, version 25.0 (IBM Corp., Armonk, NY, USA). Continuous variables were presented as mean ± standard deviation or median (minimum–maximum), whereas categorical variables were expressed as numbers and percentages. The Kolmogorov–Smirnov test was used to assess normality. Comparisons between categorical variables were performed using the chi-square or Fisher’s exact test, and continuous variables were analyzed using the Mann–Whitney U test. A two-tailed *p* value < 0.05 was considered statistically significant.

The authors used Gemini (Google) for language editing and grammatical refinement to improve the clarity and flow of the manuscript. Following this, the authors reviewed and edited the content to ensure scientific accuracy and take full responsibility for the final version of the publication.

## 3. Results

### 3.1. Patient Characteristics and Clinical Background

A total of 40 patients were included in the study. The mean age was 48 ± 13 years, with a female predominance (35, 87.5%). An atopic background was identified in 9 (22.5%) patients, primarily including allergic rhinitis (8, 20.0%) and asthma (2, 5.0%). Hypersensitivity to non-PPI drugs was reported in 9 (22.5%) cases; the most frequently implicated agents were non-steroidal anti-inflammatory drugs (NSAIDs) (3, 7.5%), beta-lactam antibiotics (2, 5.0%), and quinolones (2, 5.0%). Regarding comorbidities, metabolic or cardiovascular conditions were present in 11 (27.5%) patients, while autoimmune diseases were identified in 8 (20.0%) (Table 1).

### 3.2. Characteristics of Index Hypersensitivity Reactions

Lansoprazole was the most frequent culprit agent (20, 50.0%), followed by esomeprazole (9, 22.5%), pantoprazole (8, 20.0%), and rabeprazole (3, 7.5%). A history of reactions to more than one PPI was reported by 6 (15.0%) patients (Table 2). Regarding severity according to the Brown classification, 14 (35.0%) patients presented with Grade I reactions, 11 (27.5%) with Grade II, and 15 (37.5%) with Grade III. Consequently, systemic anaphylaxis (Grade II or III) was observed in a total of 26 (65.0%) patients.

Cutaneous symptoms were the most common findings (34, 85.0%), manifesting primarily as generalized pruritus (21, 52.5%) and urticaria (18, 45.0%). Respiratory manifestations occurred in 21 (52.5%) patients, predominantly characterized by dyspnea. Cardiovascular involvement was noted in 16 (40.0%) cases, including tachycardia (10, 25.0%) and hypotension (6, 15.0%). Neurological symptoms (10, 25.0%) primarily included altered consciousness or presyncope/syncope, while gastrointestinal manifestations were less frequent (7, 17.5%), consisting mainly of nausea and vomiting (6, 15.0%) (Table 2).

### 3.3. Diagnostic Testing Outcomes

**Figure 1:**
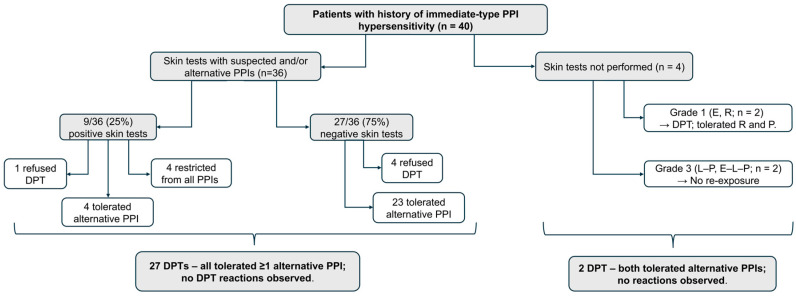
Flowchart of the diagnostic work-up and outcomes in patients with immediate hypersensitivity to proton pump inhibitors. DPT: drug provocation test; PPI: proton pump inhibitor.

The outcomes of diagnostic STs and DPTs are summarized in Figure 1 and Table 3. Diagnostic skin testing was not performed in 4 (10.0%) patients. Two of these patients (5.0%), who had a history of Grade III systemic reactions to multiple PPIs (specifically, lansoprazole–pantoprazole and esomeprazole–lansoprazole–pantoprazole), were considered at high risk for re-exposure. Therefore, all PPIs were strictly avoided, and alternative acid-suppressive therapies were initiated. The remaining 2 (5.0%) patients, who presented with Grade I cutaneous reactions to esomeprazole and rabeprazole, underwent direct oral provocation with alternative agents (rabeprazole and pantoprazole, respectively), both of which were well tolerated.

Among the 36 patients who underwent skin testing with suspected and/or alternative PPIs, 9 (25.0%) yielded positive results (Figure 1, Table 3). The positivity rates varied according to the suspected culprit agent: 31.6% (6/19) for lansoprazole, 25.0% (2/8) for pantoprazole, and 14.3% (1/7) for esomeprazole. STs were negative in both patients with suspected rabeprazole hypersensitivity. Of the six positive cases in the lansoprazole group, three reacted to lansoprazole itself, while three showed reactivity to omeprazole or pantoprazole.

In terms of clinical outcomes, among the 27 patients with negative skin tests, 23 successfully tolerated an alternative PPI via DPT, while 4 refused provocation. Of the 9 patients with positive ST results, 4 tolerated an alternative PPI, 4 were restricted from all PPIs due to within-group cross-reactivity, and 1 refused DPT (Figure 1, Table 3).

Finally, when the timing of the work-up was analyzed, the median interval between the index reaction and diagnostic testing was 7.5 months (range: 1–298 months). However, this interval was significantly shorter in patients with positive skin tests compared to those with negative results (median 2 [1–18] vs. 16 [1–298] months; *p* = 0.025).

### 3.4. Cross-Reactivity Patterns and Alternative PPI Tolerance

Consistent with the assessment criteria defined in the methods, CR was identified in 10 patients (25.0%), including cases confirmed by skin testing or DPT as well as those based solely on clinical history (Table 4). All patients in this subgroup were female, with a median age of 41 years (24–77). The median interval between the index reaction and diagnostic testing was 7.5 months (2–28).

The most frequent cross-reactivity pattern involved lansoprazole and pantoprazole (*n* = 3; Patients 6, 7, and 10). Other dual cross-reactivity patterns included esomeprazole–pantoprazole (*n* = 2; Patients 2 and 4), lansoprazole–rabeprazole (*n* = 2; Patients 3 and 8), and esomeprazole–lansoprazole (*n* = 1; Patient 1). Furthermore, broad cross-reactivity involving three distinct PPIs was observed in 2 patients: esomeprazole–lansoprazole–pantoprazole (Patient 9) and lansoprazole–omeprazole–pantoprazole (Patient 5).

Regarding the availability of safe alternatives within this cross-reactive group, 4 patients successfully tolerated a structurally different PPI: the patient with esomeprazole–lansoprazole reactivity tolerated pantoprazole; one patient with esomeprazole–pantoprazole reactivity tolerated lansoprazole; and both patients with lansoprazole–rabeprazole reactivity tolerated pantoprazole (Table 4).

### 3.5. Real-Life Follow-Up After DPT

Of the 29 patients who underwent DPT, 27 (93.1%) were successfully contacted for follow-up, while 2 could not be reached. Among these patients, 11 (40.7%) continued to use the tested PPI without any adverse events, whereas 1 (3.7%) experienced a mild Grade I reaction to rabeprazole despite a previously negative DPT result. The remaining 15 patients (55.6%) did not use a PPI during the follow-up period. For the majority of this group (*n* = 11), there was no longer a clinical indication for therapy. However, 4 patients avoided the drug despite an ongoing indication: 2 due to fear of reaction recurrence and 2 due to physician reluctance to prescribe the medication (Table 5).

## 4. Discussion

This single-center study evaluates the clinical characteristics, diagnostic outcomes, and practical implications of immediate-type HSRs to PPIs in a series of 40 patients. Our study distinguishes itself from similar reports in the literature by incorporating real-life data on patient management and drug tolerance following the diagnostic work-up.

The majority of our patients were female (87.5%), with a mean age of 48 ± 13 years. This finding is consistent with previous studies reporting that PPI hypersensitivity predominantly affects women and middle-aged individuals [4,10,11]. Approximately 22.5% of our patients had a history of atopic disease, and a similar proportion reported hypersensitivity to other drugs. This rate aligns with data from a study conducted in Türkiye, which reported an atopy prevalence of 25.8% [11]. Autoimmune, metabolic, and cardiovascular comorbidities were also observed in a subset of patients. Given that the management of these chronic conditions frequently requires long-term gastric protection, this association likely reflects increased cumulative exposure to PPIs, which is a recognized risk factor for sensitization, rather than a direct immunological predisposition.

Lansoprazole was the most frequently implicated agent, accounting for approximately half of the cases, and nearly two-thirds of patients presented with anaphylaxis. This observation aligns with previous reports indicating that PPI hypersensitivity is often associated with severe systemic reactions [4,11,12]. The predominance of lansoprazole in Türkiye [5,11], contrasting with esomeprazole and lansoprazole in Italy [4] and omeprazole in Spain [13,14], suggests that regional prescription patterns likely influence this distribution. However, limited epidemiological data and the scarcity of reports from non-European regions continue to limit a comprehensive understanding of the prevalence and global distribution of PPI hypersensitivity [2].

In addition to these epidemiological uncertainties, the diagnostic evaluation of PPI hypersensitivity presents significant challenges. In our study, skin test positivity was observed in only 25% of the 36 tested patients, corroborating the low sensitivity reported in the EAACI Position Paper [2]. Moreover, skin test positivity was significantly associated with a shorter interval between the index reaction and testing (median 2 vs. 16 months; *p* = 0.025). This finding supports the clinical relevance of performing diagnostic testing early after the reaction, as previously demonstrated by Kepil Özdemir et al. [15] and highlighted in the current EAACI Position Paper [2]. Another factor complicating the diagnostic process is the lack of commercially available intravenous formulations for certain PPIs, such as rabeprazole and dexlansoprazole [7], and the unavailability of intravenous forms of others, such as esomeprazole and lansoprazole, in many countries, including our own. This limitation significantly restricts the applicability of IDT, which is recommended by the EAACI guideline and considered a more sensitive diagnostic method. Although the basophil activation test has the potential to bridge this diagnostic gap and has been reported to show a sensitivity of 73.8% for omeprazole [14], its role in the diagnostic algorithm remains limited due to insufficient data for other PPIs and practical challenges in routine use.

Given these limitations, DPT plays a key role in confirming the diagnosis and identifying safe alternatives. In our study, all 27 patients who underwent skin testing tolerated at least one alternative PPI during DPT, demonstrating the value of this approach in avoiding unnecessary drug restrictions. Furthermore, in two carefully selected low-risk patients with a history of mild reactions, direct DPT with an alternative agent of a different chemical structure was safely tolerated, suggesting that this approach may be clinically feasible. This pragmatic strategy aligns with reports from experienced centers supporting direct DPT in low-risk patients [16]; however, it should be noted that data regarding the role and success of this approach in PPI hypersensitivity remain limited. Taken together, these findings reinforce the role of DPT not only in diagnostic confirmation but also as a practical guiding tool for risk mitigation under real-world constraints.

CR remains a major challenge in the clinical management of PPI hypersensitivity. The literature reports widespread CR among PPIs, with rates reaching up to 61.6%, and notably, approximately 8.9% of patients may exhibit CR to all available PPIs [2]. In our study, CR was detected in 10 patients (25%). While CR was confirmed by STs in 5 patients, the remaining 5 were classified as having “clinical CR” based on a strong clinical history. The lack of test confirmation in these cases reflects the multifactorial diagnostic challenges previously detailed: specifically, the unavailability of injectable forms for sensitive methods like IDT and long reaction-to-test intervals that reduce sensitivity. Furthermore, in cases with severe index reactions and no absolute indication for PPIs, our patient-safety-centered approach prioritized the avoidance of re-exposure. Therefore, these five cases are particularly valuable as they illustrate the gap between ideal diagnostic algorithms and the realities of clinical practice.

Historically, cross-reactivity among PPIs was thought to follow four general patterns based on chemical structure: sensitivity to the entire group, the omeprazole–esomeprazole–pantoprazole group, the lansoprazole–rabeprazole group, or a single PPI [12]. However, accumulating evidence suggests that this simplified classification is insufficient to explain the full clinical picture. Indeed, studies from Europe and Turkey have reported ‘atypical’ cross-reactivity combinations—such as omeprazole–lansoprazole, lansoprazole–pantoprazole, and lansoprazole–esomeprazole—that cannot be explained by this classical model [5,11,15,17,18].

Our findings further underscore the complexity of cross-reactivity in PPI hypersensitivity. We observed CR patterns aligning with chemical similarity, including esomeprazole–pantoprazole (*n* = 2) and lansoprazole–rabeprazole (*n* = 2). Conversely, we identified patterns diverging from the classical structural model, such as esomeprazole–lansoprazole (*n* = 1), and notably, the most frequent pattern in our study, lansoprazole–pantoprazole (*n* = 3). Moreover, the presence of broader CR combinations in two patients (esomeprazole–lansoprazole–pantoprazole and lansoprazole–omeprazole–pantoprazole) suggests that cross-reactivity extends beyond simple structural classifications. This heterogeneity likely stems from the intricate interplay of individual metabolic pathways, drug-related cofactors, and shared immunological determinants [2].

Our study distinguishes itself from similar diagnostic series in the literature by incorporating ‘real-life’ data obtained through prospective patient follow-up. These data, successfully collected from 27 of the 29 patients (93.1%) who underwent DPT, clearly demonstrate both the clinical validity and the limitations of the test. Eleven patients (40.7%) continued to use the safe alternative without adverse reactions, reaffirming the high negative predictive value of DPT. Conversely, a mild cutaneous reaction in one patient (3.7%) despite a negative DPT highlights that, while considered the ‘gold standard,’ this test does not offer absolute assurance.

Our most striking ‘real-life’ observation is that more than half of the patients (*n* = 15, 55.6%) did not use the alternative drug, despite DPT confirming its safety. The primary reasons were the cessation of clinical need (*n* = 11) and, even when an indication persisted, avoidance due to ‘fear of recurrence’ (*n* = 2) or ‘physician reluctance’ (*n* = 2). Specifically, avoidance driven by anxiety or physician reluctance highlights a clear gap between diagnostic confirmation and clinical practice. These findings align with the literature, which reports similar non-utilization rates (47–53%) attributed largely to the lack of clinical need and fear of re-exposure [19,20]. This underscores that DPT should be complemented by a communication process that reinforces patient education and physician confidence.

This study has several limitations. Although a sample size of 40 patients provides valuable clinical insights given the rarity of immediate-type PPI hypersensitivity, the single-center design may limit the generalizability of the findings. The retrospective collection of part of the data introduces a potential risk of recall bias, particularly regarding the interval between the index reaction and diagnostic testing. In addition, our pragmatic, patient-centered approach, in which the diagnostic work-up was discontinued once a safe alternative PPI was identified, did not allow for a complete assessment of cross-reactivity prevalence. As noted earlier, our diagnostic methodology was also constrained by real-world drug availability; the lack of injectable formulations for certain PPIs in our country precluded the systematic use of IDTs across all agents. Furthermore, the unavailability of in vitro diagnostic tools, such as the basophil activation test, restricted our ability to achieve diagnostic confirmation without in vivo provocation. Finally, the inability to reach a small number of patients during the prospective follow-up represents a minor limitation in the evaluation of real-life data.

## 5. Conclusions

Lansoprazole was the primary culprit and anaphylaxis the predominant clinical presentation. Diagnostic accuracy decreased with longer intervals between the reaction and testing, highlighting the importance of early evaluation. Crucially, drug provocation testing allowed the identification of at least one safe alternative in all tested patients, confirming its indispensable role in clinical management. However, real-life data showed that diagnostic confirmation does not always translate into drug use due to behavioral and clinician-related barriers, emphasizing the need for patient counseling and physician reassurance. Future multicenter studies are needed to better characterize cross-reactivity patterns and clarify factors influencing real-life adherence.

## Figures and Tables

**Table 1:** Demographic and clinical characteristics of patients with immediate hypersensitivity to proton pump inhibitors.

Category	Variable	Value *
Demographics	Age, years (mean ± SD)	48 ± 13
	Female	35 (87.5)
Allergic and Immunologic Background	Atopy	9 (22.5)
	Allergic rhinitis	8 (20.0)
	Asthma	2 (5.0)
	Other IgE-mediated allergy ^a^	4 (10.0)
	Chronic urticaria/angioedema	1 (2.5)
	Non-PPI drug hypersensitivity ^b^	9 (22.5)
Comorbidities	Autoimmune diseases ^c^	8 (20.0)
	Metabolic/cardiovascular diseases ^d^	11 (27.5)

* Data are presented as *n* (%) unless otherwise indicated. ^a^ Latex (*n* = 1) and venom (*n* = 3). ^b^ NSAIDs (*n* = 3), β-lactam antibiotics (*n* = 2), quinolones (*n* = 2), radiocontrast (*n* = 1), muscle relaxant (*n* = 1). ^c^ Hashimoto thyroiditis (*n* = 4), Graves disease (*n* = 3), Sjögren syndrome (*n* = 1). ^d^ Hypertension (*n* = 5), diabetes mellitus (*n* = 3), coronary artery disease (*n* = 2), obesity (*n* = 1). Abbreviations: PPI, proton pump inhibitor; SD, standard deviation; NSAID, non-steroidal anti-inflammatory drug.

**Table 2:** Culprit agents, severity, and clinical features of immediate hypersensitivity to proton pump inhibitors.

Category	Variable	Value *
Culprit PPI	Lansoprazole	20 (50.0)
	Esomeprazole	9 (22.5)
	Pantoprazole	8 (20.0)
	Rabeprazole	3 (7.5)
Recurrent HSR	Hypersensitivity to multiple PPIs	6 (15.0)
Brown classification	Grade I	14 (35.0)
	Grade II	11 (27.5)
	Grade III	15 (37.5)
Organ systems involved	Cutaneous	34 (85.0)
	Respiratory	21 (52.5)
	Cardiovascular	16 (40.0)
	Neurological	10 (25.0)
	Gastrointestinal	7 (17.5)
Baseline laboratory findings	Total IgE (IU/mL)	113.5 [5–1318]
	Baseline tryptase (µg/L)	4.38 [1.39–12.3]

* Data are presented as *n* (%) unless otherwise indicated. Laboratory values are expressed as median [minimum–maximum]. Abbreviations: PPI, proton pump inhibitor; IgE, immunoglobulin E; HSR, Hypersensitivity Reaction.

**Table 3:** Diagnostic skin test results and drug provocation test outcomes according to the suspected agent.

Suspected PPI	Positive ST Results					Successful DPT with PPI					Clinical Remarks
	L	O	P	E	R	L	O	P	E	R	
L (*n* = 19)	**3/6** ^a^	**1/1**	**3/19** ^b^	0/2	-	-	-	14/14	1/1	-	Refused DPT (P, *n* = 1) All PPIs restricted (*n* = 3)
P (*n* = 8)	0/4	0/2	**2/7**	0/1	0/1	2/2	1/1	-	1/1	1/1	Refused DPT (L, *n* = 2) Refused DPT (O, *n* = 1)
E (*n* = 7)	0/3	-	**1/6**	0/2	-	1/1	-	4/4	-	-	Refused DPT (P, *n* = 1) All PPIs restricted (*n* = 1)
R (*n* = 2)	0/1	-	0/2	-	-	-	-	2/2	-	-	-

Each superscript letter (a, b) indicates an individual patient. Bold values indicate positive skin test results. Abbreviations: L, lansoprazole; P, pantoprazole; E, esomeprazole; R, rabeprazole; O, omeprazole; DPT, drug provocation test; PPI, proton pump inhibitor; ST, skin test.

**Table 4:** Clinical characteristics and diagnostic test results of patients with cross-reactivity among proton pump inhibitors.

Patient No	Age	Grade	Test Interval Months	Culprit PPI			Skin Tests					Tolerated Alternative PPI
				1st Reaction	2nd Reaction	3rd Reaction	L	O	P	E	R	
1	39	1	4	E	L	−			−			P
2	36	2	2	E	E	−	−		+			
3	24	3	22	L	R	−			−			P
4	43	1	3	P	E	−	−		+			L
5	50	3	2	L	−	−		+	+			
6	74	3	7	L	−	−			+			
7	69	3	28	L	P	−						
8	26	1	11	R	L	−			−			P
9	29	2	25	E	L	P						
10	77	1	18	L	−	−			+			

All patients in this subgroup were female. +, positive; –, negative; blank cells indicate tests not performed. Abbreviations: L, lansoprazole; P, pantoprazole; E, esomeprazole; R, rabeprazole; O, omeprazole; PPI, proton pump inhibitor.

**Table 5:** Real-life outcomes after drug provocation testing (*n* = 27).

Real-Life Outcome After DPT	Patients *	Details
Continued PPI use without reaction	11 (40.7)	-
Reaction despite negative DPT	1 (3.7)	Rabeprazole, Grade 1
No PPI use after DPT	15 (55.6)	-
No further clinical indication	11 (40.7)	-
Avoided use despite indication	4 (14.8)	Fear of recurrence (*n* = 2) Physician reluctance (*n* = 2)

* Data are presented as *n* (%). Abbreviations: DPT, drug provocation testing; PPI, proton pump inhibitor.

## Data Availability

The data presented in this study are available on request from the corresponding author. The data are not publicly available due to privacy restrictions.

## Abbreviations

The following abbreviations are used in this manuscript:

ADR	Adverse drug reaction
CR	Cross-reactivity
DPT	Drug provocation test
E	Esomeprazole
EAACI	European Academy of Allergy and Clinical Immunology
HSR	Hypersensitivity reaction
IDT	Intradermal test
IgE	Immunoglobulin E
L	Lansoprazole
NSAID	Non-steroidal anti-inflammatory drug
O	Omeprazole
P	Pantoprazole
PPI	Proton pump inhibitor
R	Rabeprazole
SD	Standard deviation
SPT	Skin prick test
ST	Skin test
